# The Catenulida flatworm can express genes from its microbiome or from the DNA it ingests

**DOI:** 10.1038/s41598-019-55659-w

**Published:** 2019-12-13

**Authors:** Marcos Trindade Rosa, Elgion L. S. Loreto

**Affiliations:** 1 PPG Biodiverdade Animal, Univ. Fed. de Santa Maria (UFSM), Santa Maria, Brazil; 2 Department of Biochemistry and Molecular Biology, CCNE, Univ. Fed. de Santa Maria, Av. Roraima 1000, 97105-900 Santa Maria, RS Brazil

**Keywords:** Ecology, Evolution, Genetics, Molecular biology

## Abstract

*Stenostomum* are tiny planarians of the phylum Platyhelminthes that reproduce asexually. We transfected these worms using plasmids containing a *gfp* reporter gene. Here we show that they can express genes present in plasmids carried by bacteria and those that are encoded by naked DNA, such as plasmids or PCR fragments, transfected by electroporation; they can also express genes taken up during feeding. The microbiome associated with worm maintenance was evaluated, and the results indicated that when a plasmid is maintained in the microbiome, *gfp* gene expression is stable. When genes originate from naked DNA or bacteria not maintained in the microbiome, GFP expression is transient. Therefore, changes in the microbiome can modify the ability of worms to express foreign genes. In stable GFP-expressing worms, NSG showed that the *gfp* gene was maintained in the plasmid and was not integrated into the chromosome. These results suggest that, at least for some organisms such as flatworms, the expression of genes provided by the microbiome or the environment can be considered among the potential sources of phenotypic plasticity, which can have implications for evolvability.

## Introduction

The holobiont theory posits that multicellular eukaryotes are not autonomous organisms but rather biological units comprised of the organism and numerous microbial symbionts (holobiont). This postulation brings with it a current debate in evolutionary research regarding the role of the microbiome in evolution^1–3^. The hologenome consists of the nuclear genome, organelles and microbiome, and it is important for evolution because phenotypic variation can result from the interaction of products of more than one genome in a holobiont^3^.

A wide spectrum of interactions can be found in different holobionts. In some cases, the microbiome associated with organisms is a source of different materials such as nutrients, probiotics and secondary metabolites or acts in host defense, for example by degrading xenobiotics^4–6^. In others, the members of a holobiont are completely dependent on one other and, in some organisms, the microorganisms become intracellular components of the host. In several platyhelminths, annelids and molluscs, symbiotic bacteria harboured in the trophosomes are responsible for supplying energy to the holobiont^7^. The *Paracatenula* flatworm (Catenulida), which is devoid of a mouth and gut, is an excellent example of this extreme holobiont interaction. Chemoautotrophic bacteria of the genus *Candidatus* Riegeria are a fundamental symbiont in Paracatenula’s trophosomes, corresponding to half of the biomass of these worms and being primary energy source^8^. Organisms with simple body plans, having almost all of their cells in direct contact with the surrounding environment, can have a stronger interaction with the microbiome. In sponges, for example, microbial symbionts comprise up to 35% of the holobiont biomass and are fundamental providers of metabolites for sponge physiology^9^.

In this study we present evidence that the flatworm *Stenostumum*, which is also formed by many cells in direct contact with the environment, shows a host-microbe interaction not described thus far. Their cells can express genes present in plasmids carried by their native bacteria and even genes from naked DNA, such as plasmids or PCR amplicons, taken up during feeding. The flatworm *Stenostomum* belongs to the class Catenulida, a basal group of Platyhelminthes^10–12^. They reproduce asexually by paratomy, a remarkable process in which the formation of anterior structures in the median region of the body forms zooids. After complete maturation of zooids, each one detaches to form new organisms. In *S. leucops*, the paratomy process occurs in approximately 42 h at 28 °C, producing zooids containing approximately 2400 cells. These flatworms range from 0.5 to 2 mm in length^13^. The first requisite of genetic transformation, the incorporation of foreign DNA into a cell, is the entrance of DNA into the cell. Normally, eukaryotic cells do not easily accept foreign DNA, and several procedures have been used to promote this step, such as some chemical-based methods (calcium phosphate or liposomes), physical-based methods (electroporation, microinjection, particle bombardment) and viral methods^14,15^. Transfection has been described for several Platyhelminthes species using particle bombardment or electroporation of RNA or plasmids^16^. Nucleic acid entrance into the cell can lead to biological consequences, such as expression of carried ORFs. This gene expression can be stable when the foreign DNA is inserted into the genome or maintained as stable episomes. Expression can also be transient, whereby the maintenance of DNA or RNA in the cells is temporary, and the expression is lost over time^17^.

Stable genetic transformation of Platyhelminthes was obtained using a transposon-based vector in planarians^18^ and *Schistosoma*^19^. This methodology has been widely used in other organisms, in which genes of interest are inserted between the transposon terminal inverted repeats (TIRs) in a vector plasmid. The TIR sequences are recognized by a transposase enzyme encoded by a helper plasmid. Cells transfected with both plasmids can have the sequences of the vector plasmid transposed into their chromosomes^20^.

The intake of genetic material can promote a transient gene expression, observed when the gene of interest is not integrated into the host genome and is, for instance, maintained as stable episomes. For example, it has been demonstrated that the phenotypes of various invertebrates, when exposed to dietary material containing *in vitro* synthesized dsRNA, or to organisms such as bacteria, artificially expressing dsRNA, are affected by gene silencing through the RNAi process^21,22^. In these cases, a transient gene silencing occurs while the dsRNA is present into the de host cells, or the bacteria expressing dsRNA are present in the host diet.

In this study, we show that *Stenostomum* can transiently express the *gfp* gene from plasmids contained in bacteria provided in their diet or even from naked DNA such as plasmids or PCR fragments. Also, stable expression of the *gfp* gene was obtained followed by plasmid maintenance in the worms, by some symbiotic bacteria. The evolutionary implications of these findings are discussed in light of the hologenome theory.

## Results

**Figure 1:**
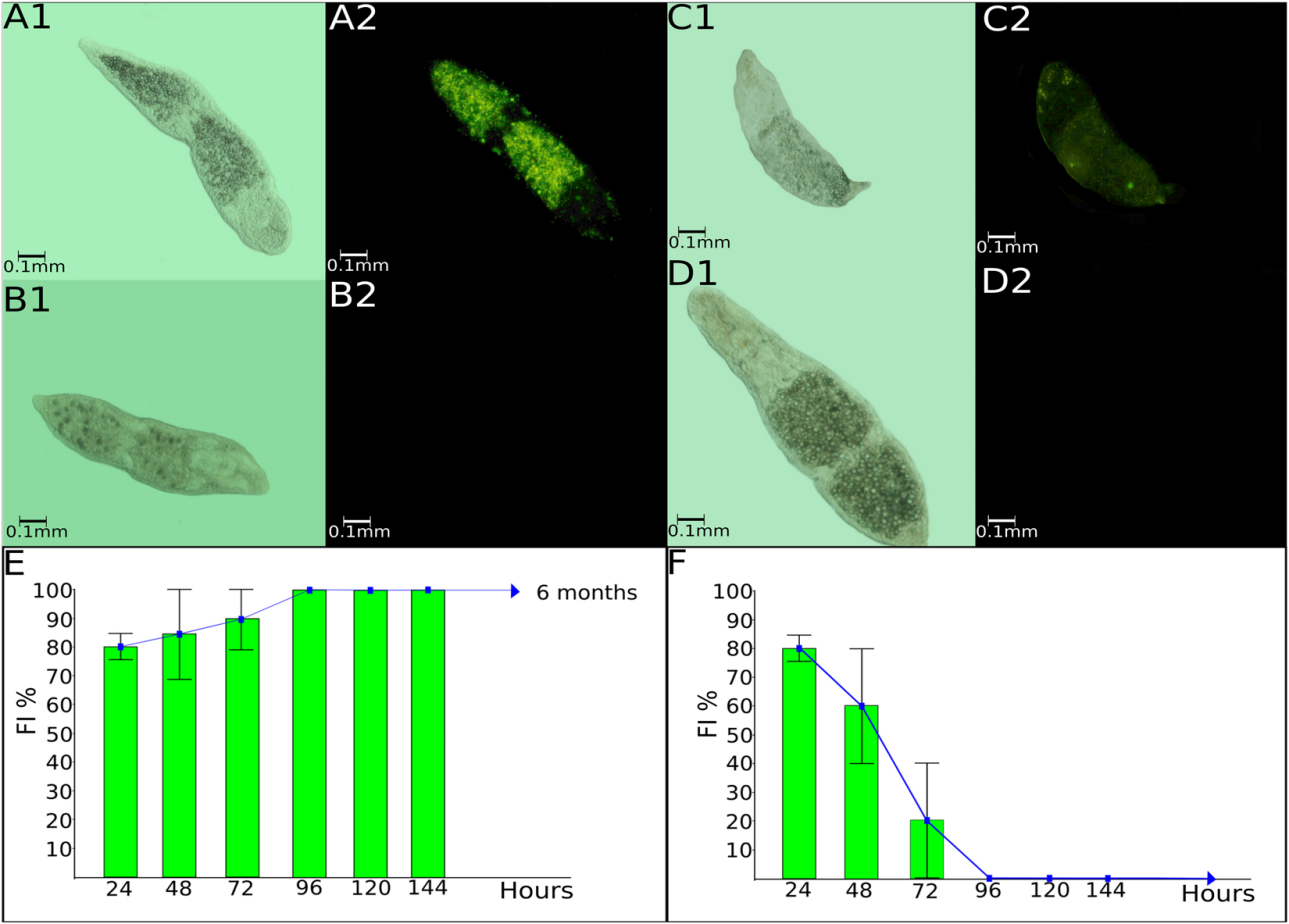
*Stenostomum leucops* transfected with pBac[3xp3-EGFPafm] and pB∆Sac plasmids. Bright-field microscopy of SL0-sm01 worms electroporated with plasmids (**A1**) and under fluorescence microscopy (**A2**), showing fluorescence; (**B1**) SL0-sm01 worm electroporated without plasmids (control) not showing fluorescence (**B2**); (**C1**) SL0-sm02 worms electroporated with plasmids and under fluorescence microscopy, showing fluorescence **(C2)**; **D1**- SL0-sm02 worm electroporated without plasmids (control) not showing fluorescence (**D2**); (**E**) percentage of fluorescent individuals (FI) for SL0-sm01 worms electroporated with plasmids, in relation to the time (hours) after electroporation; (**F**) percentage of fluorescent individuals (FI) for SL0-sm02 worms electroporated with plasmids. Eighty worms were visualized under the microscope in each assay.

Our initial goal was to promote genetic transformation of worms using a transposon vector and *gfp* as a reporter gene, as described for planarians by González-Estévez *et al*.^18^. *S. leucops* of the SL0-sm01 and SL0-sm02 strains transfected by electroporation with pBac[3xp3-EGFPafm] and pB∆Sac plasmids had survival rates of 20+/− 10% for both strains. However, GFP expression differed between tested strains (Fig. 1). For SL0-sm01, 80+/− 5% of worms showed strong GFP expression 24 hours after electroporation, reaching 100% in 96 hours. Transfected worms of this strain showed stable GFP expression, transmitted to subsequent generations for six months (see Supplementary Movie 1). The SL0-sm02 strains showed weaker and transient GFP expression. Twenty-four hours after electroporation, 80+/− 5% of worms showed GFP fluorescence. However, GFP expression decreased and was lost after 96 hours.

**Figure 2:**
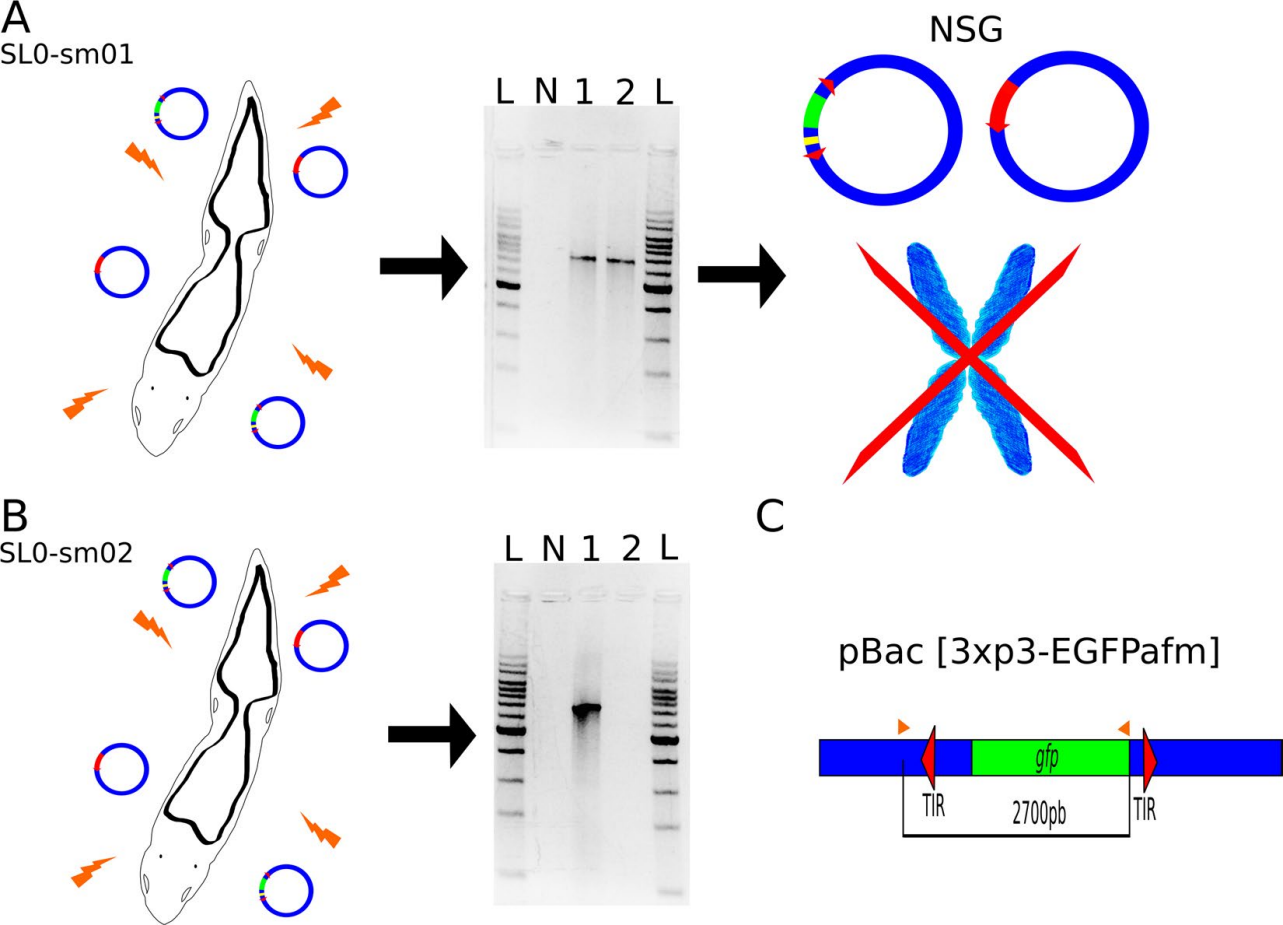
Experimental scheme testing the integration of the *gfp* gene into the *Stenostomum* genome. (**A**) SL0-sm01 electroporated worms using pBac [3xp3-EGFPafm] and pB∆Sac plasmids were tested by PCR (gel lanes: L = Kb ladder; N = control/ no DNA; 1 = worm sample three days after electroporation; 2 = worm sample 15 days after electroporation). Amplicons of 2,700 bp indicated that precise transposition had not occurred (see scheme **C**). Illumina NSG using gDNA isolated six months after electroporation allowed assembly of both plasmids (complete and circularized). However, no indication of integration into the worm’s chromosomes was obtained. (**B**) SL0-sm02 electroporated worms using pBac[3xp3-EGFPafm] and pB∆Sac plasmids tested by PCR 3 and 15 days after electroporation. No amplicons were obtained 15 days after electroporation, showing the plasmid was lost in this period; (**C**) scheme of the pBac[3xp3-EGFPafm] plasmid containing *piggyBac* TIRs sequences (red arrowhead) and *gfp* gene (green), other plasmid sequences (blue) and primers used for PCRs (orange arrows). Amplicons containing 81 bp of plasmid sequences upstream of the TIRs, suggest that a precise transposition did not occur.

Aiming to verify if stable GFP expression observed in SL0-sm01 strain was a result of transposition of this gene into the *S. leucops* genome or the result of a plasmid being maintained in the worm, PCR-based assays were performed three and fifteen days after electroporation (Fig. 2, Supplementary Table 4). In SL0-sm01 worms, amplicons containing regions outside of the TIR

sequences of the transposon vector were obtained. This suggests that, if integration into the *S. leucops* chromosomes had occurred, it was not a result of precise excision mediated by the *piggyBac* transposase (Fig. 2A,C; Supp. Fig. 1). Alternatively, the complete plasmids could be maintained in the worms. For SL0-sm02, amplicons were obtained three days after electroporation but not fifteen days after, showing that the transfected plasmids had been lost (Fig. 2B). These results are concordant with the transient GFP expression observed in SL0-sm02 strain.

To distinguish between the hypotheses of sequence integration into chromosomes and plasmid maintenance in the SL0-sm01 strain, the genome of these worms was sequenced using an NGS approach. The complete sequence of plasmids pBac[3xp3-EGFPafm] and pB∆Sac were assembled with a high coverage (58X). Approximately 2,000 reads containing sequences of the pBac[3xp3-EGFPafm] plasmid were obtained, which allowed the assemblage of the complete plasmid, including the *gfp* gene. However, among those 2,000 reads, none had “hybrid” sequences (i.e sequences belonging to both the genome of *Stenostomum* and the transfected plasmid), suggesting that no integration into the worms’ genome occurred. The assembly metrics are presented in Supplementary Table 1. The transformed animals maintained fluorescence for six months, corresponding to around 80 generations after genetic transformation. These animals belonging to the 80 generation were used for genomic DNA isolation for NGS. Therefore, the complete plasmids were maintained in the worm culture during this time.

**Figure 3:**
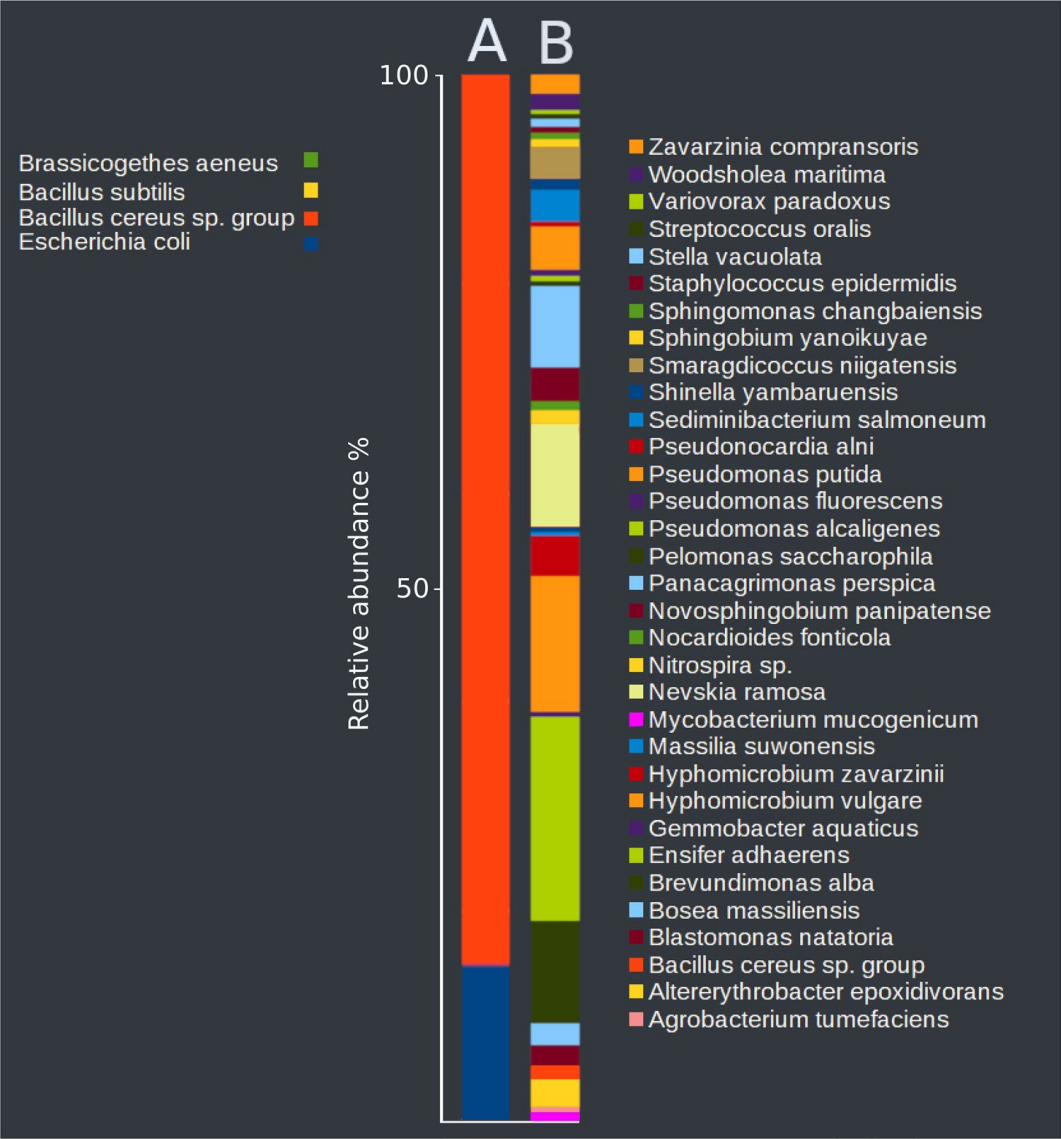
Microbiome associated with *Stenostomum leucops*: (**A**) microbiome present in the SL0-sm01 strain; (**B**) microbiome present in the SL0-sm02 strain. The bar plots represent relative abundance (in %).

Aiming to test if the different pattern of plasmid maintenance observed in SL0-sm01 and SL0-sm02 strains could be related to the microbiome associated with the worms, we analysed the microbiomes of these strains using a 16S rRNA gene amplicon NGS approach (Fig. 3). In strain SL0-sm01 four bacterial taxa were found, with *Bacillus cereus* sp. being the most abundant group (85.15%) followed by *Escherichia coli* (14.74%), *B. subtilis* (0.06%) and *B. aeneaus* (0.05%). In strain SL0-sm02 thirty-six bacteria species were found. *E. coli* was absent, and the *Bacillus cereus* species group corresponded to only 1.2% of sequenced reads. These results show that the microbiome is quite different between the strains used. More significantly, these results suggest that the plasmid could be maintained by *E. coli*, as the used plasmid has the ColE1 replication origin, which is related to this bacterium and other closely related species.

**Figure 4:**
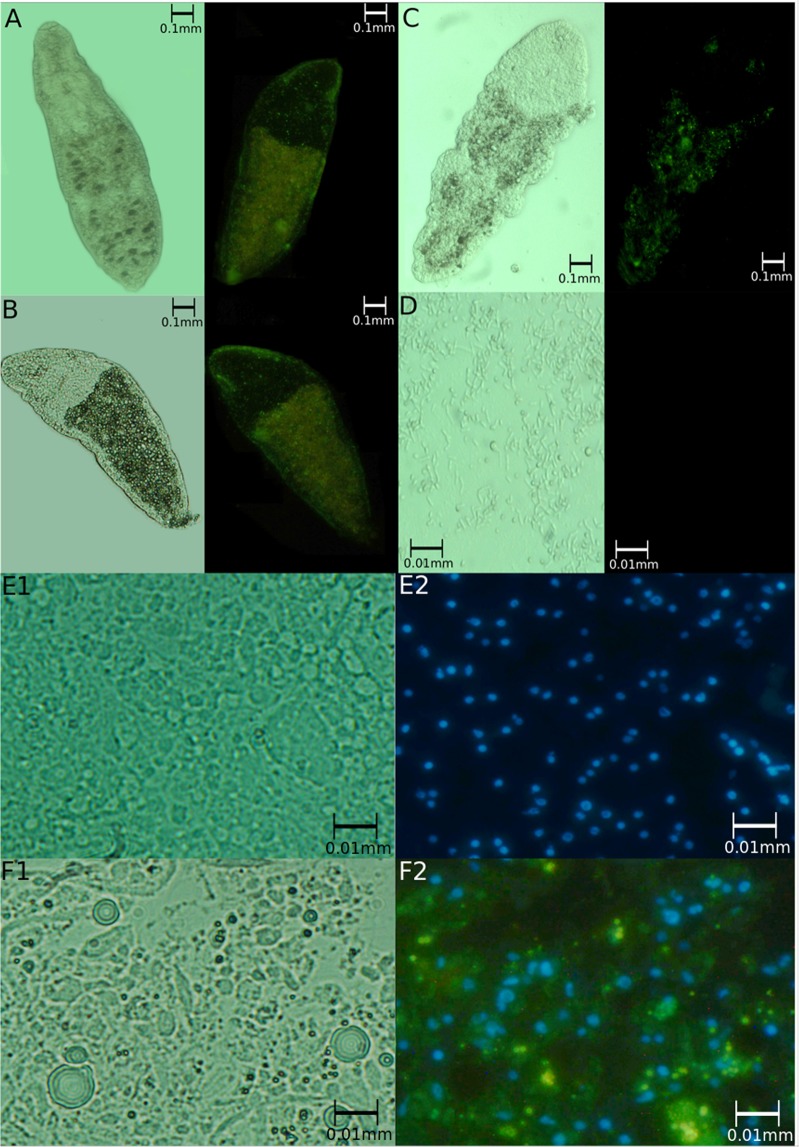
GFP expression in worms fed (**A**) *E. coli* XL1 containing pBac [3xp3-EGFPafm] plasmid; (**B**) only the pBac [3xp3-EGFPafm] plasmid; (**C**) 3.2 kb PCR fragment containing complete *gpf* gene. (**D**) the bacteria *E. coli* XL1 harbouring the pBac [3xp3-EGFPafm] plasmid; E1) *Stenostomum* cell, stained with DAPI, in bright-field microscopy; E2) in fluorescence microscopy (blue = nucleus); F1) *Stenostomum* cell of worms fed *E. coli* XL1 containing pBac [3xp3-EGFPafm] plasmid, stained with DAPI, in bright-field microscopy; F2) in fluorescence microscopy (blue = nucleus; green = GFP). Sixty worms were visualized under the microscope in each assay.

To test the hypothesis that plasmids maintained by *E. coli* could be the gene source of GFP expression, we fed the worms of SL0-sm02 strain with *E. coli* Xl1 (a laboratory strain) containing the plasmids. As can be seen in Fig. 4A, the worms fed with this bacterium expressed GFP. This expression was transient, lasting for three days. The same occurred when worms were fed with “naked DNA” as plasmid or PCR fragments containing the *gfp* gene (Fig. 4B,C), suggesting that GFP expression requires DNA containing the *gfp* gene and not the bacteria. Reinforcing this hypothesis, we showed that *E. coli* XL1 harbouring the plasmid with the *gfp* gene does not fluoresce (Fig. 4D). Instead, by disintegrating the worms, allowing isolated cells to be seen, we could observe fluorescence in the cytoplasm of cells from worms fed with plasmids containing the *gfp* gene but not in the cells of worms that did not receive the plasmids (Fig. 4E,F). These results point out that the *gfp* gene contained in DNA present in the *Stenostomum*’s feed can be expressed in the worm’s cells. In conclusion, the transfected gfp gene was shown to be transiently expressed by the worm when presented as “naked DNA” or in plasmids carried by bacterial strains that cannot be maintained as part of *Stenostomum* microbiome (such as the auxotrophic laboratory strain of *E.coli* Xl1). However, when the plasmid is harbored by native *E. coli*, the GFP expression becomes stable over time.

**Figure 5:**
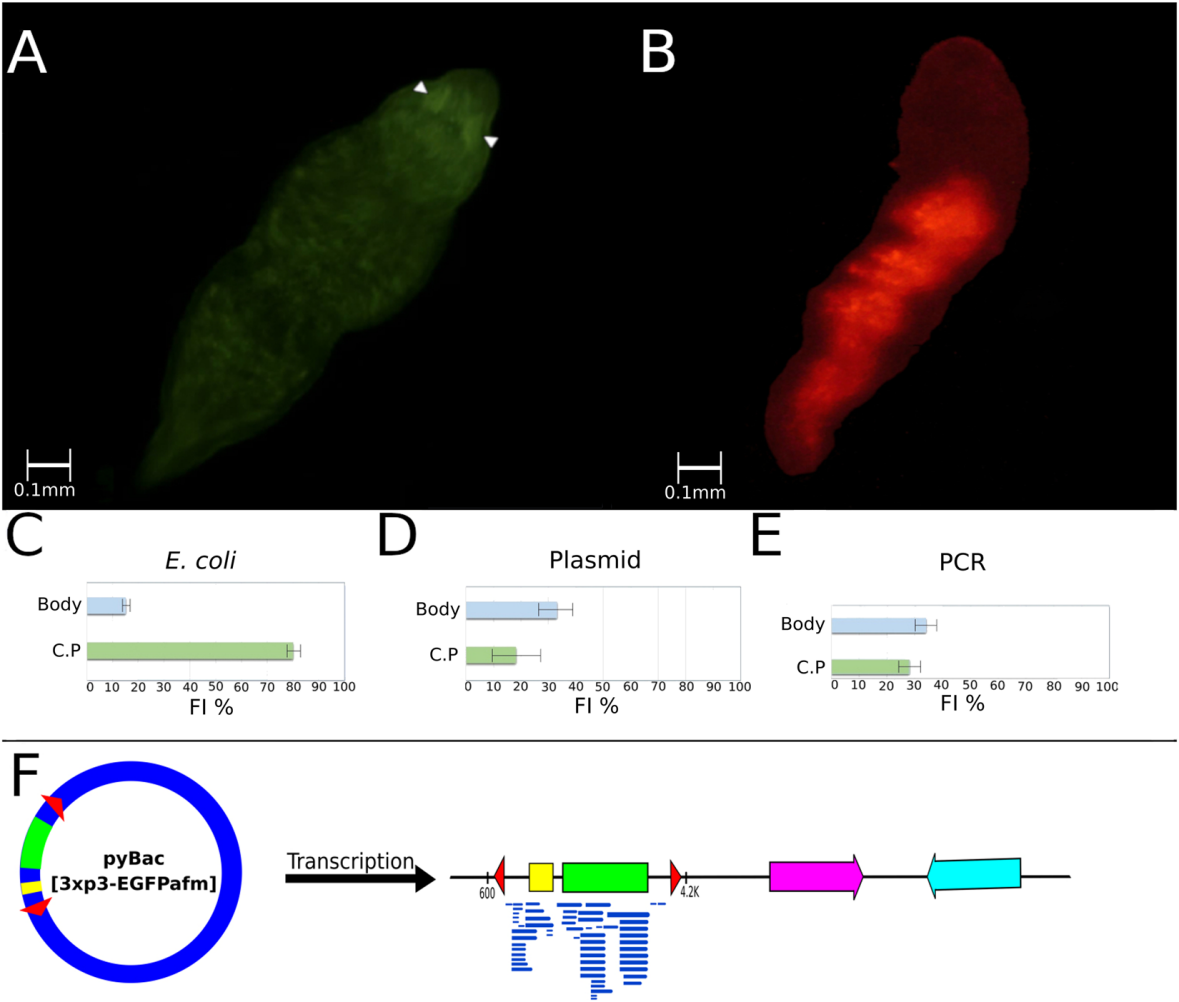
Expression of the *gfp* reporter gene under the control of different promoters. (**A**) GFP expression mainly in the ciliated pits (CPF, arrowhead) when plasmids have Pax6 regulatory elements in the promotor; (**B**) pattern of diffuse fluorescence (DF) spread over the body observed in worms expressing mCherry red fluorescent protein under control of the histone H3 promotor; (**C**) percentage of worms showing diffuse fluorescence spread over the body (blue bars) or in the ciliated pit (green bars), for worms fed with: *E. coli* carrying 3xp3-EGFPafm (with Pax6 RE) plasmid or only the plasmid (**D**); (**E**) PCR fragments containing the GFP gene with Pax6 RE (green bars). Whiskers represent the standard deviation. (**F**) Transcriptome assay for worms fed 3xp3-EGFPafm plasmid showed reads covering only the gfp gene region.

A remarkable observation was the pattern of GFP expression in *S. leucops*, in both strains tested, when the pBac [3xp3-EGFPafm] plasmid was used. The GFP expression was spread across the whole body of the worm, and more intense expression was observed in the ciliated pits (Fig. 5A). The ciliated pits are chemosensory structures, comparable to the eyespots of other planarians. The *gfp* gene used in our study is under control of a promoter with the Pax6 dimeric binding sites, and it is specifically expressed in the eye tissue of various arthropod and planarian eyespots*.* Although variable in frequency, GFP expression in the ciliated pits was observed in worms fed bacteria or “naked” DNA harbouring *gfp* gene (Fig. 5C–E). We decided to test the reporter gene under other regulatory sequences present in different plasmids. As it is demonstrated in Fig. 5B, worms harboring bacteria carrying the *mCherry* gene, under control of the Histone H3 promoter, displayed red fluorescence spread over most of their body.

A low-coverage transcriptome was assembled from worms fed with pBac [3xp3-EGFPafm] plasmid, aiming to analyse the plasmid sequences that were transcribed in the worms. Only reads covering the *gfp* gene were obtained, suggesting that only this gene was transcribed (Fig. 5F).

**Figure 6:**
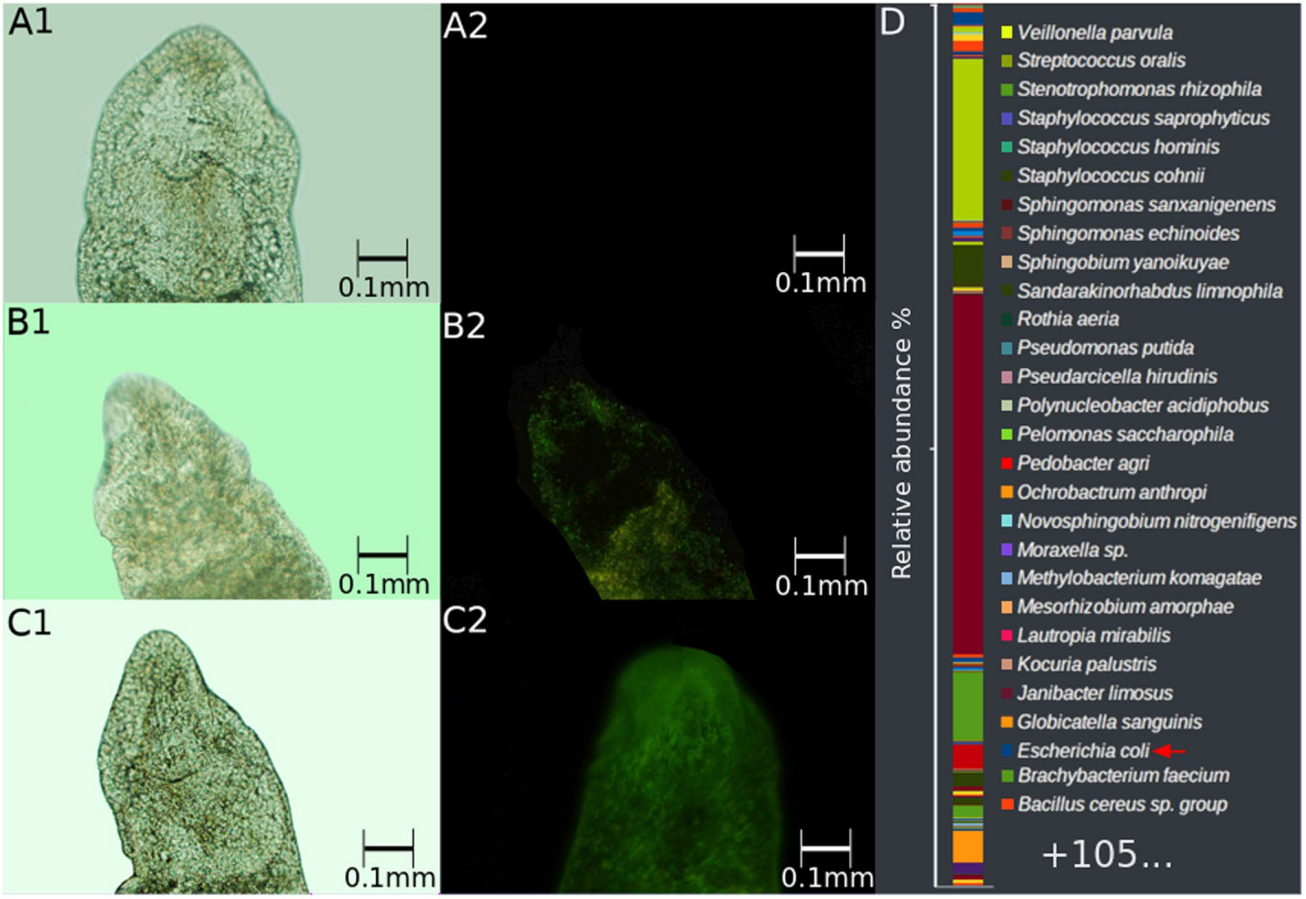
Stable GFP expression induced by the new *E. coli* strain isolated in nature, harbouring the pBac [3xp3-EGFPafm] plasmid: (**A**) Ssp-sm09, control - worms fed *E. coli* without plasmid (A1 = bright-field; A2 = fluorescence microscopy); (**B**) Ssp-sm09 fed with *E. coli* harbouring pBac[3xp3-EGFPafm] plasmid (B1 = bright-field; B2 = fluorescence microscopy); (**C**) Ssp-sm09 fed “naked” pBac[3xp3-EGFPafm] plasmid (C1 = bright-field; C2 = fluorescence microscopy); (**D**) microbiome present in the Ssp-sm09 strain. The bar plots represent relative abundance (in %).

To show that *E. coli* harbouring plasmids is able to maintain GFP fluorescence in a stable pattern and, because the laboratory strain (XL1) is auxotrophic and unable to be maintained in worms, we sought *E. coli* strains in nature that could be maintained in worms. Bacterial colonies were isolated on LB plates after electroporation of *Stenostomum sp*. Ssp-sm09, freshly collected from the wild, with the pBac[3xp3-EGFPafm] plasmid. These isolated bacteria can be maintained in a stable pattern in both worm strains, SL0-sm02 and Ssp-sm09, and the worms began to express GFP in a stable pattern, in both strains (Fig. 6A–C; Suppl. Fig. 2). These bacteria were confirmed as *E. coli* by Sanger sequencing of the ribosomal 16S sequence. Also, the microbiome of *Stenostomum* sp. Strain Ssp-sm09 was analysed and shown to be composed of 133 bacterial species, among them *E. coli* (Fig. 6D).

A comparison of the microbiomes found in the three strains of *Stenostomum* analysed showed that it was highly variable in the number and composition of species. Of a total of 156 species, only one bacterial taxon (*Bacillus cereus* sp. group) was shared by all strains. One species was shared by SL0-sm01 and Ssp-sm09 (*E. coli*), and 11 species were shared by SL0-sm02 and Ssp-sm09 (Supplementary Table 2).

## Discussion

Foreign gene expression, even from naked DNA such as a plasmid, is a well-documented phenomenon in eukaryotes. This biological property forms the basis for the promising technology of DNA vaccines, in which DNA contains a gene encoding an antigen, and a promoter/terminator is expressed in mammalian cells, inducing immunity^23,24^. However, DNA uptake is not a spontaneous process, and several transfection procedures are normally used to induce this step^14^. Several studies have shown that, in mammals, meal-derived DNA fragments of up to 300 bp can avoid degradation in the digestive system, enter the circulation system and be found in the blood^25^. The novelty we introduce with our study, at least to our knowledge, is that the worm *Stenostomum* can spontaneously take up foreign DNA provided by different routes, including by feeding, and can express the protein-coding sequences that it contains.

Transient gene expression induced by feeding on genetic material was first shown in *C. elegans* eating double-stranded RNA (dsRNA) or bacteria expressing dsRNA. These feeding materials have the ability to silence genes by dsRNA-mediated genetic interference (RNAi)^21^. Dietary exposure of several invertebrate organisms has shown similar results^22^. In Platyhelminthes, feeding the freshwater planarian *Schmidtea mediterranea* on *E. coli* expressing double-stranded RNA inhibits gene expression by RNAi^26^. These dsRNA involved in feeding-mediated RNAi are molecules <200 nucleotides in length that typically do not code for a protein. In *C. elegans*, dsRNA uptake is mediated by a channel protein (SID-1)^27^. Homologues to this protein are found in other organisms although, in several insects, SID-like proteins are not involved in dsRNA uptake. In *Drosophila*, the entrance of dsRNA into the cell involves clathrin-mediated endocytosis^28^. In summary, these studies have shown that diet-derived dsRNA is able to modulate gene expression by RNAi. However, in the present work, a different phenomenon was described, in which phenotypic expression is related to ingested DNA and not dsRNA. Also, the sizes of the fragments involved were larger than those described for dsRNA uptake, since the smallest DNA fragment we used was 3394 bp, the largest plasmids are approximately 9 kbp and the CDS contained in the ingested DNA was expressed.

The expression of foreign genes is normally transient, mainly in mitotically active cells, when organisms or cells are transfected with plasmids, DNA fragments or episomes^17^. However, more stable gene expression from plasmids has been described in the literature. For example, plasmids persisted in mouse skeletal muscle for 19 months, expressing the *luciferase* gene after intramuscular injection^29^. Similarly, we observed stable GFP expression in transfected *Stenostomum* worms, when they hold up in their microbiota. However, a difference can be highlighted: rodent muscle cells are post-mitotic, meaning they do not divide further, which can explain plasmid maintenance in these cells^29^. In contrast, *Stenostumum* is an organism that undergoes intense cell division, and stable expression was associated with the maintenance of *E. coli*. The plasmids used in the transfection had a replication origin for this species (ColE1), meaning that they can be maintained in *E. coli* and related species. The absence of *E. coli* in strain SL0-sm02 could explain its inability to stably express GFP.

The fluorescence observed in the worms could be from GFP expressed by the ingested bacteria. However, some evidence indicates that DNA uptake by worm cells is the more plausible explanation: (i) bacterial colonies grown in Petri dishes did not show fluorescence; (ii) naked DNA containing the *gfp* gene is enough to produce fluorescence; (iii) the artificial Pax-6 promoter is recognized by the transcriptional machinery of eukaryotes, but not by bacteria, and there was marked expression of GFP in the ciliated pit; (iv) fluorescence is observed in the cytoplasm of worms cells; (v) transcriptome analyses showed only transcripts of the *gfp* gene, suggesting an eukaryotic transcription pattern.

An increasing number of examples have been reported showing that some phenotypes are not the result of only one genotype but rather, the interaction of products provided by two or more genotypes, i.e. the hologenome. In some cases, remarkable relationships have evolved; for example, the Hawaiian bobtail squid has a light organ to harbour fluorescent bacteria and, using an extraordinary mechanism of molecular signalling between the squid and the endosymbiont, the two organisms are able to glow at night in a coordinated manner^30^. Furthermore, the microbiome is now known to have a significant role in metabolism, immune defense, and behaviour^31^. Symbiosis can produce selectable genetic variation for the holobiont, and it can generate reproductive isolation, which has significant evolutionary implications^32^. In sponges, which are organisms with a simple body plan, the cells are in direct contact with the surrounding medium, and the bacterial symbionts are of central importance for their metabolism and are major contributors to the evolutionary and ecological success of these organisms^9^. Catenulida worms, like sponges, have a simple body plan and cells in direct contact with the surrounding medium, which may be involved in their capacity to obtain genetic material from the environment and express it. As these materials can be provided by the microbiome, this provides a new route for holobionts to increase their arsenal of evolutionary tools. Catenulida show extraordinary phenotypic plasticity^13^. Also, their microbiome, as shown here, can be highly variable. It is worth mentioning that, in our study, only the eubacteria representative of the microbiome were characterized. Thus, the microbiome probably could be more diverse if the eukaryotic and archaeal counterparts were also add to the analysis. The possibility that worms can express genes harboured by their microbiome may contribute to their phenotypic plasticity and, therefore, to their evolvability.

## Methods

### Animals used and their maintenance

Two different *Stenostomum leucops* strains were used in this work, SL0-sm01 and SL0-sm02. They were collected in a pond at Santa Maria, Brazil (53°17′ W; 29°28′S) in 2009 and maintained in the laboratory at 28 °C, in reconstituted water and fed with powdered milk Nestle/Molico^13^. Both strains had the same clonal origin. However, after several years of culture, the original strain showed a reduction in the reproduction rate. To increase the culture vigour we collected water from a dam, centrifuged it at 112 × g for 5 minutes, and added the supernatant to the culture, aiming to improve the worms’ microbiota. The centrifugation step aimed to prevent other worms from contaminating the culture. The original strain came to be called came SL0-sm01 and the strain which received the bacteria called SL0-sm02.

A third strain, Ssp-sm09, which belongs to an undescribed *Stenostomum* species, was collected in a pond at Santa Maria, Brazil (53°17′W; 29°28′S) in 2019 and maintained in the laboratory under the same conditions used to maintain the other strains. DNA barcoding (accession number MN257507) showed that this species is closely related to *S. sthenum*.

### Plasmids and PCR fragments used

Three different plasmids were used for *S. leucops* transfection: (i) pBac [3xp3-EGFPafm]^33^ containing the *piggyBac* transposon Terminal Inverted Repeats (TIRs) and the green fluorescent protein ORF under the control of an artificial promoter containing three Pax-6 homo-dimer binding sites. This promoter was constructed to drive strong expression of the GFP protein in the eye tissue of *Drosophila* and to promote expression of this gene in other organisms, as in planarian eyespots^18^; (ii) pB∆Sac – helper^34^, which contains the *piggyBac* transposase gene, but one of its TIRs was inverted, making it unrecognizable to transposase, producing a non-mobilizable transposable element copy. However, this plasmid promotes the production of *piggyBac* transposase to mobilize sequences present in the pBac [3xp3-EGFPafm] plasmid; (iii) pjaf15_H3p-Kat-H3T plasmid containing the mCherry reporter gene under control of the *Cryptococcus neoformans* Histone H3 promotor. As a control, worms were fed *E. coli* without plasmids or with plasmids not containing *gfp* reporter genes (pCR 2.1, Invitrogen; Carlsbad, Ca, USA).

A PCR product was used for transfection, corresponding to the amplicon of 3,394 bp obtained from pBac [3xp3-EGFPafm] containing the *gfp* ORF, the 3PX3 promotor region and the *piggyBac* TIRs. Primers and PCR conditions can be found in Suppl Table 3 and Suppl. Fig. 1.

### Transfection procedures

Three different procedures were used for transfection of *Stenostomum:* (*i*) electroporation; (*ii*) feeding animals with *E. coli* XL1 harbouring the desirable plasmid or (*iii*) feeding or exposing the animal to “naked DNA” (isolated plasmid or PCR products).

During the electroporation process, the worms were exposed to a single pulse of 80 V for 1 ms, using a Gene Pulser Xcell Electroporation System (Bio-Rad, Hercules, Ca, USA) apparatus. Fifty worms were put in a 2-mm cuvette with 200 µl of reconstituted water, 35 µl of PBS and 1.5 µg of DNA. After electroporation, the worms were rinsed in reconstituted water and maintained individually in wells of a plastic cell culture plate with reconstituted water (3 ml per well). For each assay, eight independent experiments were performed.

In the second procedure, the worms were fed with *E. coli* XL1 transformed with the plasmids described above. Bacteria were grown in 2 ml of LB medium for 18 hours at 37 °C. Then, the medium was centrifuged and the pellet added to 0.1 mg of powdered milk and maintained for 3 hours at 37 °C in an oven until the mixture was completely dehydrated. After this, the dried mixture was added to 2 ml of reconstituted water, in a well of a cell culture plate containing 10 worms. For each assay, 15 independent experiments were performed.

Native *E. coli* strains were isolated from *Stenostomum sp*. Ssp-sm09, freshly collected from the wild. The worms were electroporated with the pBac[3xp3-EGFPafm] plasmid and, after homogenization, were plated on LB agar medium supplemented with 35 µg/ml of ampicilin. Bacteria obtained by this process were grown in LB medium, dehydrated in powdered milk, as previously described, and used to feed SL0-sm02 and Ssp-sm09 strains.

The third procedure consisted on directly exposing the animals to “naked DNA”. This was done by adding DNA directly into the water in which the animals were maintained (0.7 µg/ml) or adding DNA to the worms’ food. A quantity of 0.7 µg of plasmid DNA or PCR fragments was added to 1 mg of powdered milk. The mixture was incubated in an oven for three hours at 37 °C, until complete dehydration. The dried mixture was added to 2 ml of reconstituted water in a well of a cell culture plate containing 10 worms. For each assay, 15 independent experiments were performed.

The fluorescence of worms was compared across treatments and the control. The worms of this strain showed a very low background fluorescence, differing strongly from the transfected worms. GFP expression was registered using an Olympus BX41 fluorescence microscope (Olympus, Shinjuku, Japan). Pictures were taken using an absorption filter at 475 nm (blue) and an emission filter at 509 nm (green).

For cellular localization of GFP expression, the rostrum region of the worms was stained using DAPI (0,2 µg/ml in PBS) and registered under bright-field and fluorescence microscopy.

### DNA extraction and whole genome sequencing

Genomic DNA was isolated from an isoline expressing GFP. This isoline was transformed by electroporation using the plasmids pBac [3xp3-EGFPafm] and pB∆Sac – helper. Molecular analysis was performed on the 80^th^ generations after genetic transformation. DNA was isolated from a pool of 100 individuals using a previously described protocol^35^. A genomic DNA library was constructed from 200 ng of DNA using Illumina TruSeq Nano DNA kit. A MiSeq Reagent Kit v3 (600 cycles) was used for sequencing to produce paired-end reads. Sequencing was performed using an Illumina MiSeq platform by Unidade de Genômica Computacional Darcy Fontoura de Almeida/LNCC/ Brazil (BAM accession number: SAMN12500763).

Raw genomic data were processed to removal of sequencing adapters and low-quality reads using the Trim Galore^36^ tools. To assemble the target regions of the genome, i.e. the green fluorescent protein gene (*gfp*), the software MITOBIM^37^ was used with default parameters and using the *gfp* sequence as a seed.

### Microbiome analyses

For microbiome analyses, DNA was isolated as previously described. The microbiome analysis was performed by Neoprospecta (https://neoprospecta.com). Regions of the 16S ribosomal gene were amplified by PCR and sequenced with an Illumina platform, as described previously^38,39^. The primers and PCR conditions used are available in Supplementary Table 3. Bioinformatic analysis was performed to implement a blast search using a proprietary databank (Neoprospecta), which contained only 16S sequences of validated taxa^38^. The microbiome sequencing reads are available at the accession number: SAMN12561444 (*Stenostomum leucops* Sl0-sm01), SAMN12561445 *(Stenostomum leucops* Sl0-sm02) and SAMN12561446 (*Stenostomum sp*. Ssp-sm09).

The isolated bacterial strains obtained from *Stenostomum* sp. Ssp-sm09, have the 16S ribosomal gene amplified using the same primers used in microbiome analyses (Supplementary Table 3). The PCR product was marked using the BigDye kit and sequenced by using the Sanger method in an AB 3500 platform (Thermo Fischer Scientific, Waltham, Ma, USA).

### Transcriptome analyses

Aiming to verify which regions of plasmid are expressed in transfected worms, we performed a low-coverage transcriptome analysis. The *Stenostomum* sp. Ssp-sm09 strain was chosen because this species is larger, and fewer animals are necessary for library preparation. The animals were fed with powdered milk and plasmid as described in the transfection section. Twenty-four hours later, they were checked in a microscope for fluorescence expression, and total RNA was extracted from 30 worms using TRIzol (Invitrogen, Carlsbad, CA, USA) according to the manufacturer’s instructions. For library preparation, mRNA was enriched with the Dynabeads mRNA Purification kit (Invitrogen), according to the manufacturer’s protocol. Sequencing was performed in an Ion Torrent S5 sequencer (Thermo Fisher Scientific) using the Ion 540 Kit-OT2 and the Ion 540 Chip. Data were collected using Torrent Suite v4.0 software.

The assemblage of reads was performed by the *de novo* approach using Trinity^40^ software available in the Galaxy^41^ platform, with default parameters. The assembled contigs were blasted against the plasmid pBac [3xp3-EGFPafm]^33^. The reads were mapped using bowtie2^42^ and displayed using Tablet^43^ (RNA-seq accession number: SAMN12497666).

## Supplementary information

Supplementary Information

Supplementary Movie
