# Supplementary material for: The Catenulida flatworm can express genes from its microbiome or from the DNA it ingests
Source: Sci Rep. 2019 Dec 13;9:19045. doi: 10.1038/s41598-019-55659-w (PMC6910973; doi:10.1038/s41598-019-55659-w)
Supplement: Supplementary file 1 — Supplementary Information [file 41598_2019_55659_MOESM1_ESM.pdf]

Supplementary Material

The Catenulida flatworm can express genes from its microbiome or from the DNA it ingests

Supplementary movie1 – A comparison between *Stenostomum leucops* SL0-sm02 that consumed bacteria harbouring plasmids with *gfp* gene and worms that have ingested bacteria without the plasmids, under bright-field and fluorescence microscopy.

Supplementary Table 1 - Summary statistics of plasmids assembly

Plasmid	Assemblage	Length bp	Reads used	Mismatch	Max. coverage	Average coverage
pBac [3xp3-EGFPafm]	1 contig (circularized)	8,876	1969	4.1	152	57.961
pBΔSac	1 contig (circularized)	5,906	1368	3.8	147	48.781

The *S. leucops* genome was sequenced using the Illumina MiSeq platform and generated 13,643,983 reads with size among 35-301 bp. After trimming, it was filtered 10,583,723 reads with size among 50-300 bp, these data were used for the plasmids assemblage. Both plasmids (pBac [3xp3-EGFPafm] and pBΔSac) were assembled complete and circularized.

Supplementary Table 2 - Summary of microbiome associated with the *Stenostomum* strains used in this study: *Stenostomum leucops* SL0-sm01 and SL0-sm02; *Stenostomum sp.* Ssp-sm09. The results are expressed in % of reads. In blue are highlighted the bacterial species shared among the three strains; in red between SL0-sm02 and Ssp-sm09; in yellow between SL0-sm01 and Ssp-sm09.

	SL0-sm01	SL0-sm02	Ssp-sm09
Bacteria species	% of reads	% of reads	% of reads
Acidovorax delafieldii	0	0.82	0.06
Acidovorax soli	0	0	0.28
Acinetobacter johsonii	0	0	0.38
Acinetobacter lwoffii	0	0	0.03
Acinetobacter ursingii	0	0	0.50
Actinomyces oris	0	0	0.06
Actinomyces viscosus	0	0	0.06
Aeromonas hydrophila	0	0	0.03
Agrobacterium tumefaciens	0	0.49	1.28
Altererythrobacter epoxidivorans	0	2.70	3.54
Arthrobacter ureafaciens	0	0	0.03
Aureimonas jatrophae	0	0	0.03
Bacillus cereus sp. group	85.02	1.23	0.22
Bacillus oshimensis	0	0	0.06
Bacillus subtilis	0.06	0	0
Blastomonas natatoria	0	1.96	0
Bosea massiliensis	0	2.13	0
Bosea thiooxidans	0	0	0.09
Brachybacterium faecium	0	0	0.06
Brachybacterium sacelli	0	0	0.13
Bradyrhizobium elkanii	0	0	0.22
Bradyrhizobium genosp. SA-1	0	0	0.19
Brassicogethes aeneus	0.05	0	0
Brevibacterium aureum	0	0	0.16
Brevibacterium epidermidis	0	0	0,03
Brevibacterium iodinum	0	0	0,03
Brevibacterium pityocampae	0	0	0,03

Brevibacterium senegalense	0	0	0,03
Brevundimonas alba	0	9.82	0
Brevundimonas subvibrioides	0	0	0,13
Brevundimonas vesicularis	0	0	0,03
Candidatus Prevotella conceptionensis	0	0	0,03
Caulobacter sp.	0	0	1,44
Caulobacter vibrioides	0	0	0,03
Chryseobacterium viscerum	0	0	0,03
Corynebacterium accolens	0	0	0,78
Corynebacterium afermentans	0	0	0,06
Corynebacterium coyleae	0	0	0,06
Corynebacterium durum	0	0	0,03
Corynebacterium fastidiosum	0	0	0,03
Corynebacterium imitans	0	0	0,03
Corynebacterium jeikeium	0	0	0,03
Corynebacterium mucifaciens	0	0	0,06
Corynebacterium singulare	0	0	0,38
Corynebacterium thomssenii	0	0	0,13
Corynebacterium tuberculostearicum	0	0	0,41
Cryobacterium arcticum	0	0	0,03
Cryobacterium psychrotolerans	0	0	1,69
Cupriavidus metallidurans	0	0	0,09
Curvibacter delicatus	0	0	0,16
Dermabacter hominis	0	0	0,06
Devosia insulae	0	0	2,82
Dietzia psychralcaliphila	0	0	0,06
Dietzia timorensis	0	0	0,22
Dolosigranulum pigrum	0	0	0,03
Dyadobacter fermentans	0	0	0,03
Dyadobacter soli	0	0	2,74
Empedobacter falsenii	0	0	0,03
Ensifer adhaerens	0	19.48	0
Enterobacter falsenii	0	0	0,03
Enterobacter aerogenes	0	0	0,03
Escherichia coli	14.73	0	0.03
Finegoldia magna	0	0	0,03
Gemmobacter aquaticus	0	0.49	0
Gemmobacter lanyensis	0	0	0,03
Globicatella sanguinis	0	0	0,03
Haemophilus parainfluenzae	0	0	0,06
Hyphomicrobium vulgare	0	12.93	0,28
Hyphomicrobium zavarzinii	0	3.85	0
Janibacter limosus	0	0	0,19
Kingella oralis	0	0	0,03
Kocuria kristinae	0	0	0,03
Kocurria palustris	0	0	0,03
Lactobacillus acidophilus	0	0	0,03
Lactococcus lactis	0	0	0,06
Lautropia mirabilis	0	0	0,03
Leifsonia xyli	0	0	0,03
Massilia haematophila	0	0	0,03

Massilia suwonensis	0	0.41	0
Mesorhizobium amorphae	0	0	0,13
Mesorhizobium loti	0	0	0,06
Methylobacterium hispanicum	0	0	0,03
Methylobacterium komagatae	0	0	0,5
Methylobacterium radiotolerans	0	0	0,34
Methyloversatilis universalis	0	0	0,03
Moraxella sp.	0	0	0,03
Mycobacterium mucogenicum	0	0.49	0
Nevskia ramosa	0	9.74	40,68
Nitrospira sp.	0	1.39	0
Nocardioides fonticola	0	0.82	0
Novosphingobium aromaticivorans	0	0	0,03
Novosphingobium nitrogenifigens	0	0	0,03
Novosphingobium panipatense	0	3.27	0
Novosphingobium soli	0	0	0,03
Novosphingobium subterraneum	0	0	0,13
Ochrobactrum anthropi	0	0	0,06
Ochrobactrum pseudogrignonense	0	0	0,03
Panacagrimonas perspica	0	7.77	0
Pantoea ananatis	0	0	0,03
Pedobacter agri	0	0	0,03
Pedomicrobium americanum	0	0	0,06
Pelomonas puraquae	0	0	0,28
Pelomonas saccharophila	0	0.41	0,22
Peptoniphilus gorbachii	0	0	0,03
Phenylobacterium murchangponense	0	0	0,06
Polynucleobacter acidiphobus	0	0	4,58
Porphyrobacter donghaensis	0	0	0,41
Porphyrobacter neustonensis	0	0	0,5
Pseudarciella hirudinis	0	0	0,09
Pseudomonas alcaligenes	0	0.49	0
Pseudomonas fluorescens	0	0.65	0,06
Pseudomonas pseudoalcaligenes	0	0	0,56
Pseudomonas putida	0	4.17	0,38
Pseudonocardia alni	0	0.41	0
Ralstonia pickettii	0	0	0,5
Rhizobium nepotum	0	0	0,06
Rothia aeria	0	0	0,06
Rothia amarae	0	0	0,13
Rubrobacter xylanophilus	0	0	0,03
Sandarakinorhabdus limnophila	0	0	0,06
Sediminibacterium salmoneum	0	3.03	18,27
Shinella yambaruensis	0	1.15	0
Smaragdicoccus niigatensis	0	2.95	0
Serratia marcenscens	0	0	0,28
Sphingobium yanoikuyae	0	0.74	0,06
Sphingomonas canadensis	0	0	0,13
Sphingomonas changbaiensis	0	0.65	0
Sphingomonas cynarae	0	0	0,03
Sphingomonas echinoides	0	0	0,38
Sphingomonas leidyi	0	0	1,19

Sphingomonas melonis	0	0	0,69
Sphingomonas sanxanigenens	0	0	0,03
Sphingomonas sp.	0.06	0	0
Staphylococcus aureus	0	0	0,03
Staphylococcus capitis	0	0	0,13
Staphylococcus cohnii	0	0	0,06
Staphylococcus epidermidis	0	0.57	0,56
Staphylococcus haemolyticus	0	0	0,03
Staphylococcus hominis	0	0	0,06
Staphylococcus pasteuri	0	0	0,16
Staphylococcus pseudintermedius	0	0	0,06
Staphylococcus saprophyticus	0	0	1,35
Staphylococcus warneri	0	0	0,47
Stella vacuolata	0	0.74	0
Stenotrophomonas maltophilia	0	0	0,06
Stenotrophomonas rhizophila	0	0	0,06
Streptococcus australis	0	0	0,03
Streptococcus macedonicus	0	0	0,03
Streptococcus oralis	0	0.49	0,13
Streptococcus salivarius	0	0	0,09
Streptococcus sanguinis	0	0	0,09
Uncultured Oscillatoria sp.	0	0	0,03
Variovorax paradoxus	0	0.41	0
Veillonella parvula	0	0	0,03
Woodsholea maritima	0	1.47	0,03
Zavarzinia compransoris	0	1.88	0

Supplementary Table 3 – Primers used, PCR parameters

Amplification	Primers	Parameters	Amplicon size
plasmid pBac [3xp3-EGFPafm] / *gfp* gene and flanking sequences<br><br>(see Supplementary Figure 1)	pyBACqPCR_F: CAATCTCACAGACAG CGTTGACAATCTCAC<br><br>pyBACqPCR_exc CGACTTGGTTTGCCA TTCTT<br><br>ygfpR_Elg TTACTTGTACAGCTCT CGTCCATGC<br><br>ygfpF_Elg ATGGTGAGCAAGGGC GAG	95°C-5 min<br>[95°C-1min<br>58°C-30sec<br>72°C-2min: 30 sec]X35<br>72°C-7 min<br>4°C-∞	3394 bp<br>2700bp<br>723bp<br><br>(see Supplementary Figure 1)
Bacterial 16S V3-V4 ribosomal gene (rRNA)	341F CCTACGGGRSG CAGCAG<br><br>806R GGACTACHVGGGTW TCTAAT	95°C for 5 min<br>[95°C for 45s,<br>55°C for 30s and<br>72°C for 45s] x35<br>72°C for 2 min	305bp

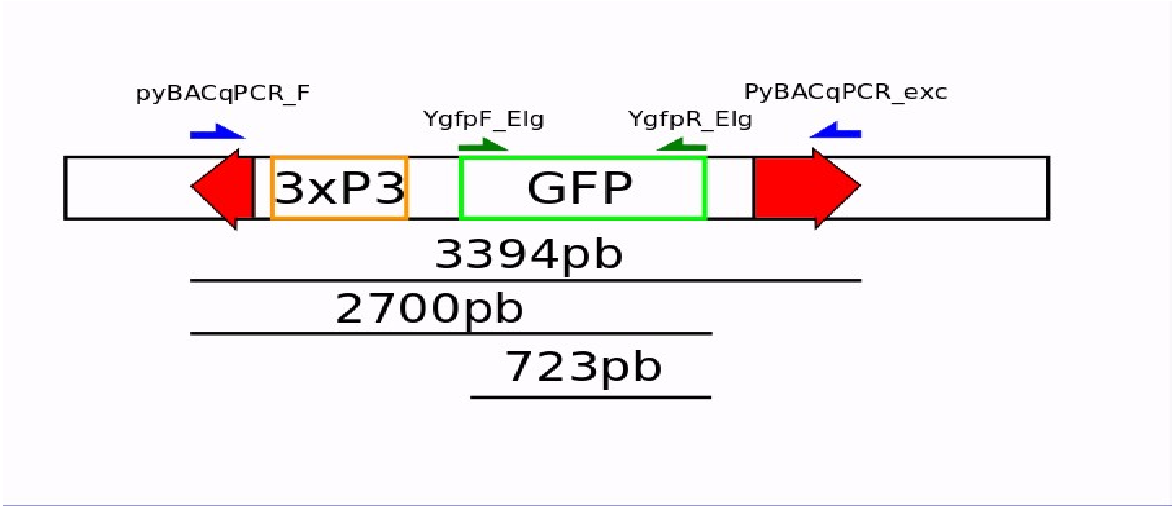

Supplementary Figure 1: Schematic representation of primers used and amplicons produced in PCR assays, testing the presence of pBac [3xp3-EGFPafm] plasmid, in worms fed with bacteria harbouring this plasmid.

Supplementary Table 4: Results of PCR assays, using different set of primers, for SL0-sm01 and SL0-sm02 worms fed with *E. coli* harbouring pBac [3xp3-EGFPafm] plasmid, 3 and 15 days after of the bacterial ingestion

PCR primers/amplicon size	SL0-sm01		SL0-sm02	
	3 days after	15 days after	3 days after	15 days after
pyBACqPCR_F - PyBACqPCR_exc 3394 pb	+	+	+	-
pyBACqPCR_F - ygfpR_Elg 2700 pb	+	+	+	-
YgfpF_Elg - ygfpR_Elg 723 pb	+	+	+	-

Supplementary Figure 2- Long term maintenance of wild *E. coli* bacteria in *Stenostomum leucops* SL0-sm02 strain

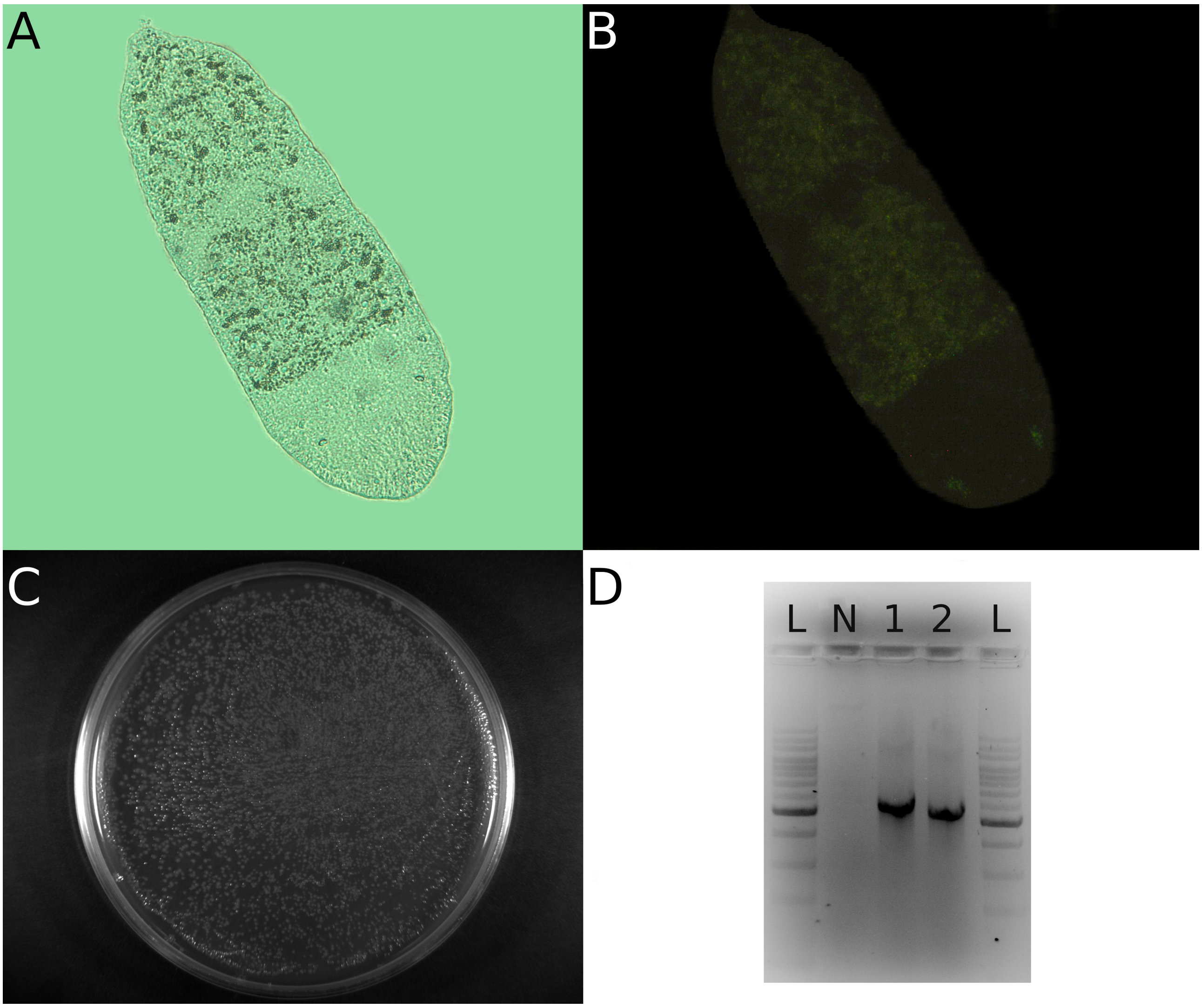

Supplementary Figure 2- maintenance of native *E. coli* bacteria in the *S. leucops*.
Stenostomum leucops SL0-sm02 strain three months after receive wild *E. coli* bacteria harbouring the pBac [3xp3-EGFPafm] plasmid. These worms were fed only once with this bacterium, which is thereafter kept in the worm microbiome. A) Bright-field microscopy of SL0-sm02 worm; B) under flourescence microscopy; C) worms were mechanically disintegrated and plated in a LB medium with ampicillin antibiotic. As can be seen, *E. coli* bacteria were maintained (confirmed by Sanger sequencing of rRNA 16 gene; five bacterial colonies had de DNA sequenced); D) PCR assay showing amplification of a region of pBac [3xp3-EGFPafm] plasmid (L = Kb ladder; N = control- none DNA template; 1= amplicom -2.7kb- generated using DNA extracted from a colony of bacteria obtained three months after feeding the worms with bacteria harbouring the plasmid; 2 = positive control, DNA of pBac [3xp3-EGFPafm] plasmid used as template). Fifteen bacterial colonies were tested by PCR and showed amplicons similar to the one showed in lane 1.
